# Pediatric Circumcision in the 21st Century National Health Service: A Snapshot of Practice in a United Kingdom Center

**DOI:** 10.1055/s-0040-1721430

**Published:** 2020-12-14

**Authors:** Patrick Jones, Helen Rooney, Amr Hawary

**Affiliations:** 1 Department of Urology, Great Western Hospital, Swindon, United Kingdom

**Keywords:** circumcision, balanitis xerotica obliterans, phimosis, pediatrics, surgery

## Abstract

**Introduction** Pediatric circumcision is a commonly performed operation, yet outcomes related to procedures performed for medical indications remain underreported.

**Aim** The aim of this study was to report outcomes of therapeutic circumcision from our center.

**Methods** Prospective registry of elective circumcisions was maintained and analyzed at a single institution in the United Kingdom. Data collected included information on complications (early and late), emergency presentations, and referrals back from primary care services.

**Results** Between August 2015 and June 2019, 300 patients (mean age: 9 years; range: 3–16 years) underwent therapeutic circumcision. The average length of follow-up data available was 2.1 years (range: 6 months to 4 years). The overall complication rate was 4.7% ( *n* = 16). There were no unplanned admissions and no cases returned to the operating room as emergency. Only 1% ( *n* = 3) of patients presented with an early complication (minor bleeding, pain, urinary retention), and 3.7% ( *n* = 11) suffered a late complication (meatal stenosis [2.7%]). All cases of meatal stenosis had lichen sclerosus confirmed on histology. Cosmetic satisfaction was 99%.

**Conclusion** Therapeutic circumcision is an effective procedure in the pediatric population, which carries a low risk of early and late complications. Our study found that meatal stenosis only occurred in those patients with confirmed lichen sclerosus histology.

Pediatric circumcision is a commonly performed operation, and it is estimated that 20.8% of males in the United Kingdom have undergone the procedure. 1 There is a large body of outcome data; however, most of the available literature is predominantly related to procedures performed for nontherapeutic male circumcision (NTMC). 2 3 Such procedures in newborns are performed to yield a lifelong reduction in the risk of medical conditions caused by the foreskin, 4 as well as disease prevention. The principle reasons why this procedure is opted for includes health, hygiene, and cosmesis. At the current time in England, circumcision can only be performed in the National Health Service (NHS) when done for therapeutic reasons.

Outcomes of circumcisions performed in the hospital setting for medical indications are underreported. 5 6 While it is considered a minor surgical procedure, there are several associated complications and adverse sequelae. These include meatal stenosis, recurrent infection, and urethral fistula. 7 8 9 Complication rates have been recorded as 10 to 20 times higher in older age groups compared with infancy. 3

Up to date, clinical evidence is therefore paramount to allow for clear patient counselling and aid the consent process with the child and his family. 10 Given the known litigious issues surrounding male circumcision, awareness and availability of up to date published outcomes related to this surgery when performed for medical indications are particularly important. 10 11 12 The aim of this study was to report outcomes of therapeutic circumcision from our center.

## Materials and Methods

Over a 4-year period (2015–2019), a prospective database was maintained at our hospital for all patients (≤16 years of age) undergoing elective circumcision for clinically diagnosed pathological phimosis. Inclusion criteria were those cases performed for indications of clinically diagnosed lichen sclerosus and recurrent balanoposthitis as per the local Clinical Commissioning Group (CCH) guidelines. All patients should have been treated with conservative measures including a trial of steroid cream application where indicated.

The electronic database of the hospital allowed any subsequent attendance(s), such as to the emergency department (ED) or urgent care presentations to be reviewed, as well as any kind of specialist referral that was made. Case notes for patients were then reviewed. Outcomes of interest included, but were not limited to, early (≤30 days) and late complications (>30 days). The former were categorized according to the Clavien–Dindo classification system. 13 Information was also collected on baseline demographics and comorbidities.

All the surgical procedures were performed by a single surgeon using the same modified guillotine technique for each case. Postoperative instructions were provided, as well as topical antibiotic ointment, regular analgesia, and emergency contact details. The information pack included directions and advice on cleaning, showering, and resting. All patients were followed up in the outpatient setting 3 months following surgery.

## Results

Between August 2015 and June 2019, 300 patients (mean age: 9 years; range: 3–16 years) underwent therapeutic circumcision. While the vast majority of patients were otherwise fit and well, 2.7% (8/300) had a comorbidity. All cases were discharged the same day and there were no unplanned or overnight admissions. The average length of follow-up data available was 2.1 years (range: 6 months to 4 years). The overall complication rate was 3.7% ( *n* = 11). Of the 300 patients, 99% ( *n* = 297) were satisfied with cosmetic appearance at follow-up.

### Early Complications

Only 1% ( *n* = 3) of patients presented with an early complication, and these all represented the 1% ( *n* = 3) of cases, which attended the ED ( Table 1 ). These adverse events were all minor (Clavien ≤ 2) in nature and resolved without surgical intervention. No major complications (Clavien ≥ 3) and no deaths were recorded. No patients were readmitted to the hospital as inpatients, and none returned to the operating theater. One patient presented with minor bleeding as a result of a loose stitch, which settled with reapplication of dressing only. One patient was brought to the ED by parents due to discomfort. This was relieved with simple analgesia alone. Finally, one patient returned with postoperative acute urinary retention, which resolved with conservative management.

**Table 1:** Summary of complications

Complication	% ( *n* )	Intervention
Early (<30 days)		
Bleeding	0.3 (1)	Conservative
Pain	0.3 (1)	Simple analgesia
Urinary retention	0.3 (1)	Conservative
Late		
Meatal stenosis	2.7 (8)	Meatoplasty
Overall complication rate	3.7%	

### Late Complications

Of the patients, 2.7% ( *n* = 8) required subsequent meatoplasty for meatal stenosis, which was the commonest postoperative complication recorded. Of note, one patient had undergone primary meatoplasty at the time of circumcision. In all these cases, lichen sclerosus ( *balanitis xerotica obliterans* ) was confirmed on histology.

## Discussion

### Key Findings

The results of this study support a low rate of complications associated with therapeutic circumcision in the pediatric population (early 1%, late 2.7%). This includes <1% of patients suffering minor bleeding, postoperative pain, and urinary retention. No cases were returned to the operating room on an emergency basis. Overall, 2.7% required meatoplasty at a later date, and all these cases had lichen sclerosus confirmed on histology at original surgery.

Meatal stenosis represents one of the important discussion points when counselling for this procedure. 14 Several theories exist to explain the possible cause(s). These include ischemia as a result of injury to frenular blood supply and meatitis due to irritants, for example, ammonia in wet nappies. 15 16 17 In a recent meta-analysis by Morris et al, which investigated the rates of postcircumcision surgery meatal stenosis, it was less than 1% in the general population but between 10 and 20% in patients affected by lichen sclerosus, which is much more common in uncircumcised males. 18 In our study, specimens were sent for histological analysis in select cases only. This was performed in the most severe cases as determined at the time of the surgery, and early application of topical steroid cream was prescribed accordingly. All the cases that went on to have meatoplasty had confirmed pathological lichen sclerosus diagnosed from the original surgery. In all these cases, the operation notes clearly documented the narrow, typically pin-hole appearance of the meatus. It is therefore difficult from our findings to determine if the original surgery itself was the cause of the meatal stenosis or whether it was all due to the underlying lichen sclerosus, which caused the presenting pathological phimosis. While it is common in many centers for histology to be performed in adult circumcision due to the risk of penile cancer, there is a lack of consensus in pediatric setting. 19 20 Green et al analyzed data from nearly 8,000 cases of patients with suspected lichen sclerosus and no cases of malignancy were identified. 21 In a study of 460 patients by Pearce et al, none of the patients had their later management changed on the basis of original histology. 22 This study also a recorded cost of £34.33 for each specimen analyzed.

In 15.7% ( *n* = 47) of the initial consultation letters, the surgeon had highlighted the strong wish raised by the family for the child to have partial or hemicircumcision only. While the surgeon had then clearly discussed and outlined the reason for standard technique, the authors believe this is an interesting observation, which highlights the strong cultural overlay, which can be present and the public's desire for the “perfect” cosmetic result. 23 24 To aid patient satisfaction, there have been efforts to deliver improved surgical techniques. This has included use of sutureless methods for tissue approximation such as with glue. 25

## Limitations

This is an observational study, and the findings are reported from a single center only. However, the data have been collected prospectively and it befits a more in-depth review of any postoperative events, which is often not possible in population-based national studies, such as those using Hospital Episode Statistics (HES) data. Analysis of HES data for example is susceptible to coding error and selection bias. While studies do report outcomes of circumcisions with much larger numbers, these are for routine circumcisions with nonmedical indications and usually in the adult setting. Contemporary literature on therapeutic circumcision in the pediatric setting is more limited, yet it is an important topic, which can cause much anxiety to parent(s) and child. Our study is one of the largest and most up to date series reported from a United Kingdom center, and these data could be incorporated in the counselling and consent process for surgery. However, we have provided our rationale for this practice. Future studies could consider implementation of visual analog scales for the assessment of pain and/or cosmetic satisfaction.

### Global Perspectives on Nontherapeutic Male Circumcision

NTMC attracts considerable debate and discussion, and at present there exists no universal consensus regarding it. However, a body of evidence now exists, which supports its value as a possible health prevention method similar to public health efforts such as childhood vaccination. 3 A recent risk analysis of United Kingdom data by Morris et al found that benefits exceed risk by 200 to 1. The potential for cost savings is another important consideration. Kacker et al highlighted the economic implications of declining NTMC trends. 26 They determined that if the rate of male circumcision in the United States decreased to 10% (54.7% in 2010), there would be an associated increase in lifetime health care costs of US$407 and US$43 for males and females, respectively.

## Conclusion

Therapeutic circumcision is an effective procedure in the pediatric population, which carries a low risk of early and late complications. The early and late complication rate in this study was 1 and 3.7%, respectively. Our study found that meatal stenosis only occurred in those patients with confirmed lichen sclerosus histology.
